# Comparison of *Enterococcus faecium* and *Enterococcus faecalis* Strains Isolated from Water and Clinical Samples: Antimicrobial Susceptibility and Genetic Relationships

**DOI:** 10.1371/journal.pone.0059491

**Published:** 2013-04-01

**Authors:** Gonzalo Castillo-Rojas, Marisa Mazari-Hiríart, Sergio Ponce de León, Rosa I. Amieva-Fernández, Raúl A. Agis-Juárez, Johannes Huebner, Yolanda López-Vidal

**Affiliations:** 1 Programa de Inmunología Molecular Microbiana, Departamento de Microbiología y Parasitología, Facultad de Medicina, Universidad Nacional Autónoma de México, México City, México; 2 Laboratorio de Ecología Química, Instituto de Ecología, Universidad Nacional Autónoma de México, México City, México; 3 Subdirector de Servicios Paramédicos, Instituto Nacional de Ciencias Médicas y Nutrición “Salvador Zubirán”, México City, México; 4 Division of Infectious Diseases, Department of Medicine, University Medical Center Freiburg, Freiburg, Germany; U. S. Salinity Lab, United States of America

## Abstract

Enterococci are part of the normal intestinal flora in a large number of mammals, and these microbes are currently used as indicators of fecal contamination in water and food for human consumption. These organisms are considered one of the primary causes of nosocomial and environmental infections due to their ability to survive in the environment and to their intrinsic resistance to antimicrobials. The aims of this study were to determine the biochemical patterns and antimicrobial susceptibilities of *Enterococcus faecalis* and *E. faecium* isolates from clinical samples and from water (groundwater, water from the Xochimilco wetland, and treated water from the Mexico City Metropolitan Area) and to determine the genetic relationships among these isolates. A total of 121 enterococcus strains were studied; 31 and 90 strains were isolated from clinical samples and water (groundwater, water from the Xochimilco wetland, and water for agricultural irrigation), respectively. Identification to the species level was performed using a multiplex PCR assay, and antimicrobial profiles were obtained using a commercial kit. Twenty-eight strains were analyzed by pulsed-field gel electrophoresis (PFGE). *E. faecium* strains isolated from water showed an atypical biochemical pattern. The clinical isolates showed higher resistance to antibiotics than those from water. Both the enterococci isolated from humans, and those isolated from water showed high genetic diversity according to the PFGE analysis, although some strains seemed to be closely related. In conclusion, enterococci isolated from humans and water are genetically different. However, water represents a potential route of transmission to the community and a source of antimicrobial resistance genes that may be readily transmitted to other, different bacterial species.

## Introduction

The genus *Enterococcus* is characterized by individual, paired, or short-chain gram-positive catalase-negative cocci. Enterococci emerged in the last decade of the twentieth century as one of the primary causes of hospital-acquired infections, although enterococci can also cause human infections in the community [1]. Enterococci can survive in a variety of environments, such as soil, water, food, plants, and animals [2], [3], [4]. In humans, as well as in other mammals and birds, these microbes are mainly found in the gastrointestinal tract as commensals but may become opportunistic pathogens in individuals with serious diseases whose immune systems are compromised and in patients who have been hospitalized for prolonged periods or who have received broad-spectrum antimicrobial therapy. Enterococci possess intrinsic or acquired resistance to several antimicrobials, such as glycopeptides, β-lactams, and fluoroquinolones, and can exhibit high levels of resistance to aminoglycosides (gentamicin and streptomycin), leading to drastically reduced therapeutic options for patients infected with enterococci, such that these bacteria are regarded as important pathogens with clinical relevance [5].

Enterococcus transmission occurs endogenously (through translocation from the gut to the bloodstream) and exogenously (in the hospital environment, e.g., via inanimate objects and the hands of health care workers and visitors) or through the consumption of contaminated food and water, the latter being the most common route of transmission, especially in developing countries [6], [7]. The Mexico City Metropolitan Area (MCMA) is a critical region from an environmental point of view, and the availability of clean water in this region is one of the most important issues for the maintenance and future development of the urban ecosystem. In the MCMA, the aquifer recharge and groundwater extraction and distribution systems are susceptible to contamination, and water quality varies seasonally. The presence of enterococci is predominantly reported during the dry season and is associated with fecal contamination [8].

The biochemical characteristics, antimicrobial susceptibility and molecular typing results of clinical and environmental *E. faecalis* and *E. faecium* isolates have not yet been reported for Mexico. The biochemical patterns and the antimicrobial susceptibility, as well as the genetic relationships between human and environmental *E. faecalis* and *E. faecium* isolates, will provide valuable information relevant to the epidemiology of infections caused by enterococci. Pulsed-field gel electrophoresis (PFGE) has been shown previously to be an excellent and highly reproducible method to identify clonal relationships among isolates [1], [9], [10], [11]. Therefore, the purpose of the present study was to determine the origins of *E. faecalis* and *E. faecium* strains isolated from hospitals, groundwater designated for human usage, water from the Xochimilco wetland, and treated wastewater used for agricultural irrigation in the MCMA.

## Materials and Methods

### Bacterial Strains

A total of 121 *Enterococcus* strains were studied: a) 31 (25.6%) strains were isolated as part of standard care (from blood, cerebrospinal fluid, eyes, livers, peripheral venous catheters, pleural fluid, sputum, urine, wounds, and the respiratory system) at five hospitals located in the southern part of the MCMA, and b) 90 (74.4%) strains were isolated from water; of these 90 strains, eight were isolated from groundwater for human use and consumption (wells), 72 were isolated from the Xochimilco wetland (created by a historical canal system), and 10 were isolated from treated wastewater from an important treatment plant. The 90 isolates from water were collected during both the rainy (45 strains) and dry (45 strains) seasons.

### Presumptive Identification by Standard Biochemical Testing

The isolation of *Enterococcus* strains from clinical samples was performed using sheep blood agar as previously described [1]. The environmental strains were isolated using a standard membrane filtration method using K-F agar for streptococci according to procedures described by the American Public Health Association (APHA) [12]. In both cases, characteristic colonies of *Enterococcus* spp. were presumptively identified based on Gram staining, the catalase test, the hydrolysis of esculin in the presence of bile, and growth in brain-heart infusion broth containing 6.5% NaCl [1]. All isolates were kept in BHI broth with 15% glycerol at −70°C until further analysis.

### Identification Using a Commercial Kit

Presumptive *Enterococcus* spp. were identified to the species level, and their antimicrobial susceptibility profiles were established using a semi-automated MicroScan system for the identification for gram-positive cocci (Positive Combo-12. Dade, Behring). The antibiotics tested by micro-dilution in broth were ampicillin (Am), ciprofloxacin (Cp), erythromycin (E), nitrofurantoin (Fd), imipenem (Imp), norfloxacin (Nxn), penicillin (P), rifampin (Rif), tetracycline (Te), and vancomycin (Va), with the additional detection of high-level resistance to gentamicin (HLR-G) and streptomycin (HLR-S). Glycopeptide resistance tests were performed according to the CLSI 2008 guidelines [13]; vancomycin resistance (VR) was confirmed using BHI agar plates containing 6 µg/mL of vancomycin, and a teicoplanin (TEC) resistance assessment was performed by agar dilution using Mueller-Hilton agar plates containing 4, 8, 16, and 32 µg/mL of the antimicrobial agent.

### Identification by Multiplex PCR

Cell pellets of *Enterococcus* spp. were suspended in 180 µl of lysis buffer (20 µg/ml lysozyme, 20 mM Tris-HCl, 2 mM EDTA, 1.2% Triton X-100) and incubated at 37°C for 30 min, after which 20 µl of proteinase K was added to each reaction, and chromosomal DNA was extracted according to the manufacturer's instructions (QIAamp DNA Mini Kit, QIAGEN). For each strain, the DNA concentration was estimated using a NanoDrop spectrophotometer (ND-1000, Thermo Scientific), and DNA integrity was verified using a 0.8% agarose gel. A multiplex PCR assay was used to confirm the identification of *E. faecalis* and *E. faecium* strains as described previously by Layton *et al*. [14].

### Pulsed-field Gel Electrophoresis

From among the clinical and environmental isolates identified as *E. faecium* and *E. faecalis*, 28 strains were selected randomly (considering origin, rainy or dry season, and antimicrobial susceptibility pattern). Nine of the selected strains came from three hospitals located in southern Mexico City, two strains came from groundwater (wells), 16 came from surface water in the Xochimilco wetland (Xochimilco canal), and one came from treated wastewater used for agricultural irrigation (treatment plant). These strains were collected during both the dry and rainy seasons. The PFGE patterns were obtained according to a protocol using the *Sma*I restriction enzyme as previously described [15]. The restriction pattern analyses were based on published criteria [16].

### Statistical Analyses

The antimicrobial susceptibility patterns were analyzed for descriptive purposes based on relative frequencies. Comparisons of the antimicrobial susceptibility patterns of the clinical and environmental isolates were performed using the Fisher-Freeman-Halton exact test for contingency tables. All analyses were performed using Stata statistical software (Intercooled Stata 7.0 for Windows 98/95/NT Package, 2002) and StatXact (v. 4.0.1, Cytel Software Corp., 1999).

## Results

**Figure 1:**
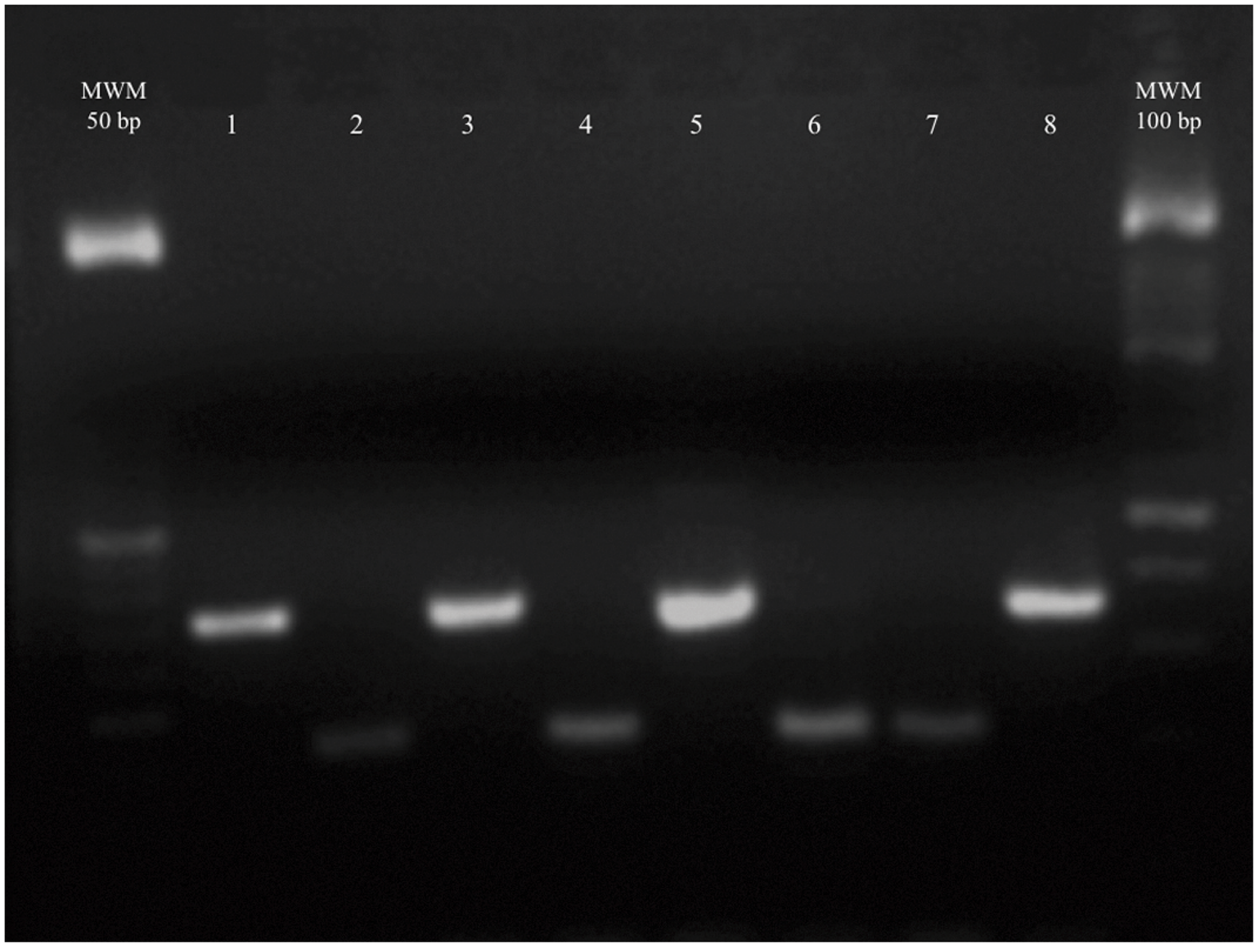
Multiplex PCR assay patterns for *E. faecium* and *E. faecalis*. Lane 1) *E. faecalis* (other respiratory); lane 2) *E. faecium* (other respiratory); lane 3) *E. faecalis* (wetland, rainy season); lane 4) *E. faecium* (water treatment plant); lane 5) *E. faecalis* (water treatment plant); lane 6) *E. faecium* (wetland, rainy season); lane 7) *E. faecium* EF1 (positive control); and lane 8) *E. faecalis* ATCC 29212 (positive control).

When the identification of the *Enterococcus* spp. was performed to the species level using both a commercial kit and a multiplex PCR method (Figure 1), we found that 27 strains had results from the commercial kit and the multiplex PCR assay that were discordant. Table 1 shows that 13 strains identified by the commercial kit as *E. faecium* were identified as *E. faecalis* by multiplex PCR analysis. In addition, three strains identified by the commercial kit as *E. faecalis* were identified as *E. faecium* using the multiplex PCR analysis. Nine strains identified by the commercial kit as *E. casseliflavus, E. durans/hirae*, *E. raffinosus* and *Enterococcus* spp. were identified as *E. faecium* and *E. faecalis* by the multiplex PCR method. Finally, two strains identified as *E. faecium* gp and as *E. faecalis* by the commercial kit were classified as *Enterococcus* spp. by the multiplex PCR assay. Given the previously reported greater specificity of the multiplex PCR assay, the results obtained using this method were used for the subsequent analysis [14].

**Table 1:** Strains with discordant results from the multiplex PCR assay and a commercial kit.

Source	Identification	
	Multiplex PCR	Commercial KIT
Water treatment plant	*E. faecalis*	*E. faecium*
Groundwater (well)	*E. faecalis*	*E. faecium*
Groundwater (well)	*E. faecalis*	*E. faecium*
Wetland	*E. faecalis*	*E. faecium*
Wetland	*E. faecalis*	*E. faecium*
Wetland	*E. faecalis*	*E. faecium*
Wetland	*E. faecalis*	*E. faecium*
Wetland	*E. faecalis*	*E. faecium* gp
Wetland	*E. faecalis*	*E. faecium* gp
Wetland	*E. faecalis*	*E. casseliflavus*
Wetland	*E. faecalis*	*E. faecium*
Wetland	*E. faecalis*	*E. durans/hirae*
Wetland	*E. faecalis*	*E. durans/hirae*
Wetland	*E. faecalis*	*E. raffinosus*
Wetland	*E. faecium*	*E. durans/hirae*
Wetland	*E. faecium*	*E. durans/hirae*
Blood	*E. faecalis*	*E. faecium*
Blood	*E. faecalis*	*E. faecium*
Other respiratory	*E. faecalis*	*E. faecium*
Urine	*E. faecalis*	*Enterococcu*s spp
Urine	*E. faecium*	*E. faecalis*
Cerebrospinal fluid	*E. faecium*	*Enterococcus* spp
Cerebrospinal fluid	*E. faecium*	*Enterococcus* spp
Catheter	*E. faecium*	*E. faecalis*
Eye	*E. faecium*	*E. faecalis*
Wetland	*Enterococcus* spp	*E. faecium* gp
Wetland	*Enterococcus* spp	*E. faecalis*

Of the 121 enterococci studied by multiplex PCR, 102 (84.3%) were identified as *E. faecium* and *E. faecalis*; of these, 48.1% (49/102) were *E. faecium* and 51.9% (53/102) were *E. faecalis.* In total, 28.6% (14/49) of the *E. faecium* came from clinical samples and 28.6% (14/49) and 42.8% (21/49) were from water collected during the rainy and dry seasons, respectively. In total, 32.1% (17/53) of the *E. faecalis* isolates were clinical isolates, and 39.6% (21/53) and 28.3% (15/53) of the *E. faecalis* isolates were from water collected during the rainy and dry seasons, respectively.

Among the isolates, 19 (15.7%) strains from environmental water samples were identified using the Dade MicroScan system as *E. durans/hirae* (10/19, 52.6%), *E. casseliflavus* (4/19, 21%), *E. gallinarum* (2/19, 10.5%), *E. raffinosus* (1/19, 5.3%), *E. faecium* gp (1/19, 5.3%), or *E. faecalis* (1/19, 5.3%). These species were not included in the subsequent analysis because our study focused only on the clinically relevant species, i.e., *E. faecium* and *E. faecalis.*

The biochemical profiles of the *E. faecium* and *E. faecalis* strains are shown in Table 2. Some of these isolates performed atypical biochemical reactions, and this pattern was particularly common among the *E. faecium* and *E. faecalis* strains isolated from water. The atypical results for the water isolates included the utilization of sorbitol, arabinose, and raffinose; positive Voges-Proskauer reaction; growth in the presence of crystal violet; and alkaline phosphatase and pyrrolidonyl arylamidase activity. Interestingly, 15 (42.8%) of the *E. faecium* strains isolated from water samples were positive for the utilization of raffinose; in contrast, 6 (17.1%) *E. faecium* strains that were also isolated from water samples and four (28.6%) that were isolated from clinical samples were positive for the use of sorbitol (Table 2). Fewer of the *E. faecalis* strains isolated from water and clinical samples were able to use sorbitol.

**Table 2:** Biochemical profiles of *E. faecium* and *E. faecalis* strains from clinical and water samples.

Characteristics	No. positive strains^a^			
	*E. faecium*		*E. faecalis*	
	Clinical^b^ 14 (%)	Water^c^ 35 (%)	Clinical 17 (%)	Water 36 (%)
Growth in crystal violet	4 (28.6)	17 (48.6)	12 (70.6)	30 (83.3)
Voges-Proskauer reaction	12 (85.7)	32 (91.4)	17 (100)	35 (97.2)
Alkaline phosphatase	3 (21.4)	2 (5.7)	14 (82.4)	22 (61.1)
Pyrrolidonyl arylamidase	14 (100)	32 (91.4)	17 (100)	32 (88.9)
Acid from:				
Mannitol	14 (100)	31 (88.6)	16 (100)	34 (94.4)
Sorbitol	4 (28.6)	6 (17.1)	13 (76.5)	15 (41.7)
Arabinose	12 (85.7)	29 (82.8)	4 (23.5)	12 (33.3)
Inulin	0 (0)	0 (0)	0 (0)	2 (5.6)
Raffinose	0 (0)	15 (42.8)	1 (5.9)	10 (27.8)
Pyruvate utilization	3 (21.4)	0 (0)	12 (70.6)	22 (61.1)

a All strains hydrolyzed esculin and grew in 40% bile and 6.5% NaCl.

b Isolated strain from blood, cerebrospinal fluid, eyes, livers, peripheral venous catheters, pleural fluid, sputum, urine, wounds, and the respiratory system).

c Isolated strains from groundwater for human use and consumption (wells), water from the Xochimilco wetland, and water from a water treatment plant (used for agricultural irrigation).

Of the strains identified as *E. faecalis* and *E. faecium*, 90.2% (92/102) were resistant to at least one antibiotic. The antimicrobial susceptibility tests showed that the clinical strains had, in general, a higher rate of resistance against the tested antimicrobials than did the isolates obtained from water samples (Table 3), particularly among the *E. faecium* strains (Table 4). We identified 34 different antimicrobial susceptibility patterns; furthermore, some specific resistance patterns were common among the clinical isolates and among the water isolates from both seasons (rainy and dry), (Table 4). Two *E. faecium* strains isolated from the Xochimilco wetland and two strains isolated from the water treatment plant during the dry season showed an intermediate level of resistance to vancomycin (Va) (8 µg/mL), as did one *E. faecalis* isolate from the Xochimilco wetland (rainy season) and one from the water treatment plant (dry season), as shown in Table 3. The clinical *E. faecium* (42.8% and 50%) and *E. faecalis* (29.4% and 41.2%) isolates showed high-level resistance to gentamicin and streptomycin, respectively (Table 3), but only two *E. faecium* strains and one *E. faecalis* strain isolated from water showed high-level resistance to streptomycin and gentamicin, respectively. All strains were susceptible to teicoplanin (data not shown). Comparing the antimicrobial patterns of the clinical and environmental isolates, we found four antibiotics that showed non-significant differences for *E. faecium* strains (Fd, Imp, Rif, and Va) and five for *E. faecalis* strains (Amp, Fd, Imp, Rif, and Va) (Table 3).

**Table 3:** Percentages of antibiotic resistance for the *E. faecium* and *E. faecalis* isolates from clinical and water samples.

Species	Origin (n)^b^	Interpretivecriteria	Antibiotic (percentage)^a^							
			Amp	Fd	Imp	Rif	Te	Va	HLG	HLS
*E. faecium*	Clinical (14)	Susceptible	42.8	92.8	100	42.8	14.3	100	57.2	50
		Intermediate	0	7.1	0	14.3	7.1	0	0	0
		Resistant	57.1	0	0	42.8	78.6	0	42.8	50
	Water (35)	Susceptible	97.1	94.3	100	62.8	82.8	88.6	100	94.3
		Intermediate	0	2.8	0	2.8	0	11.4*	0	0
		Resistant	2.8	2.8	0	34.3	17.2	0	0	5.7
		§p	0.0000	**0.63**	**NS**	**0.21**	0.0001	**1.0**	0.0001	0.0001
*E. faecalis*	Clinical (17)	Susceptible	88.2	100	94.1	58.8	35.3	100	70.6	58.8
		Intermediate	0	0	0	11.8	0	0	0	0
		Resistant	11.8	0	5.9	29.4	63.7	0	29.4	41.2
	Water (36)	Susceptible	100	97.2	100	61.1	75	94.4	97.2	100
		Intermediate	0	2.8	0	13.9	0	5.6**	0	0
		Resistant	0	0	0	25	25	0	2.8	0
		§p	**NS**	**NS**	**0.41**	**1.0**	0.0011	**NS**	0.0332	0.0076

a Interpretive criteria are according to CLSI, 2008 (Clinical and Laboratory Standards Institute, 2008); Amp = ampicillin, Fd = nitrofurantoin, Imp = imipenem, Rif = rifampin, Te = tetracycline, Va = vancomycin, HLG = high-level resistance to gentamicin, and HLS = high-level resistance to streptomycin.

b The numbers in parentheses are the numbers of strains tested.

* Four and **two strains had intermediate resistance to vancomycin (8 µL/mL). Clinical samples (blood, cerebrospinal fluid, eye, liver, peripheral venous catheter, pleural fluid, sputum, urine, wound, and respiratory system). Water samples (groundwater for human use and consumption (wells), water from the Xochimilco wetland and water from a water treatment plant (water used for agricultural irrigation).

§ Fisher-Freeman-Halton exact test. NS = not significant.

**Table 4:** Antimicrobial susceptibility patterns of *E. faecium* and *E. faecalis* isolates from clinical and water samples.

Antimicrobial susceptibility pattern	n a	*E. faecium*							*E. faecalis*						
		Clin	Rainy season			Dry season			Clin	Rainy season			Dry season		
			WL	GW	WTP	WL	GW	WTP		WL	GW	WTP	WL	GW	WTP
Amp, Cp, E, Nxn, P, Rif, Te	3	3													
Cp, E, Fd, Nxn, P, Rif, Te	1	1													
Amp, Cp, E, Nxn, P, Te	3	2							1						
Amp, E, Nxn, P, Rif, Te	1	1													
Amp, Fd, Nxn, P, Rif, Te	1					1									
Cp, E, Fd, Nxn, Rif, Te	1				1										
Cp, E, Fd, Nxn, Te	1									1					
Cp, E, Nxn, Rif, Te	2		1						1						
Amp, E, Nxn, Rif	1	1													
Amp, E, P, Rif	1								1						
Cp, E, Nxn, Te	3	2							1						
Cp, E, P, Te	1	1													
E, Imp, Nxn, Te	1								1						
E, Nxn, Rif, Te	2	1							1						
E, Nxn, Te, Va*	1					1									
E, Rif, Te, Va*	1							1							
Nxn, Rif, Te, Va*	1					1									
Amp, Nxn, Rif	1	1													
Cp, Nxn, Rif	1													1	
Cp, E, Nxn	4					3			1						
E, Nxn, Rif	1						1								
E, Nxn, Te	1								1						
E, Rif, Te	5								2	1			2		
E, Rif, Va*	2							1							1
Cp, Nxn	4		2							2					
E, P	1								1						
E, Rif	11			2		1		1	2		1		4		
E, Te	6	1							3	1			1		
Rif, Te	1									1					
Te, Va*	1									1					
E	20		1			5		1	1	9			3		
Nxn	2		2												
Rif	5					2				2			1		
Te	1												1		
TOTAL 34	92	14	6	2	1	14	1	4	17	18	1	0	12	1	1

Amp = ampicillin, Cp = ciprofloxacin, E = erythromycin, Fd = nitrofurantoin, Imp = imipenem, Nxn = norfloxacin, P = penicillin, Rif = rifampin, Te = tetracycline, and Va = vancomycin.

a Numbers of strains. Clin = Clinical samples (blood, cerebrospinal fluid, eye, liver, peripheral venous catheter, pleural fluid, sputum, urine, wound, and respiratory system). WL = water from the Xochimilco wetland, GW = groundwater for human use and consumption (wells), WTP = water from a water treatment plant (used for agricultural irrigation).

* Strains with intermediate vancomycin resistance.

**Figure 2:**
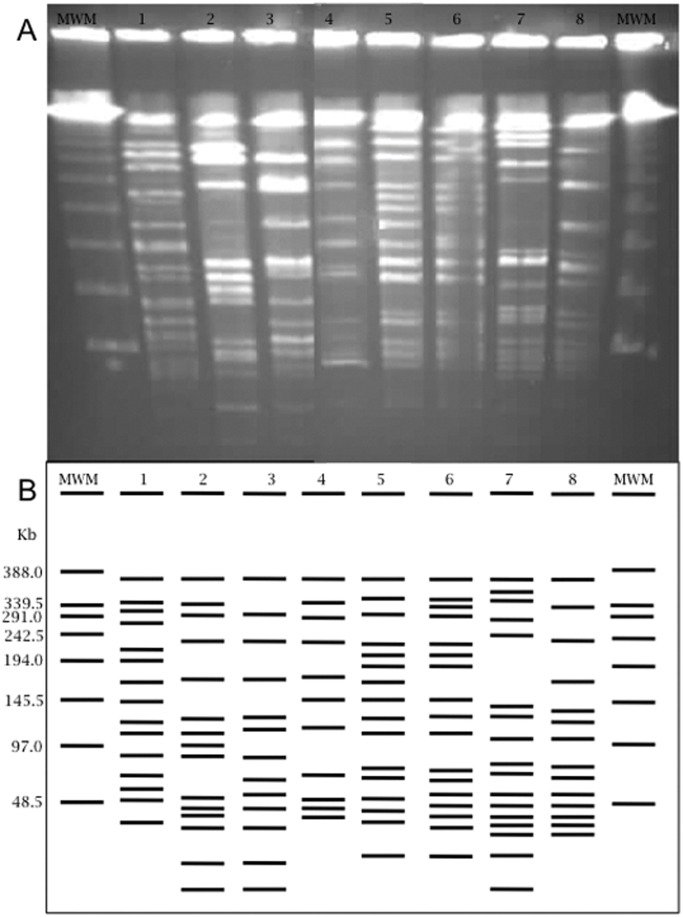
Electrophoretic PFGE patterns from *E. faecium* and *E. faecalis*. A) Agarose gel electrophoresis showing the *Sma*I digestion patterns of enterococci. B) Graphical representation of the banding patterns. Lane 1) *E. faecium* (blood); lane 2) *E. faecalis* (pleural fluid); lane 3) *E. faecalis* (urine); lane 4) *E. faecalis* (wound); lane 5) *E. faecalis* (wetland, rainy season); lane 6) *E. faecalis* (wetland, dry season); lane 7) *E. faecalis* (wetland, dry season); and lane 8) *E. faecium* (wetland, dry season). MWM = Lambda Ladder PFG Marker (New England BioLabs).

**Figure 3:**
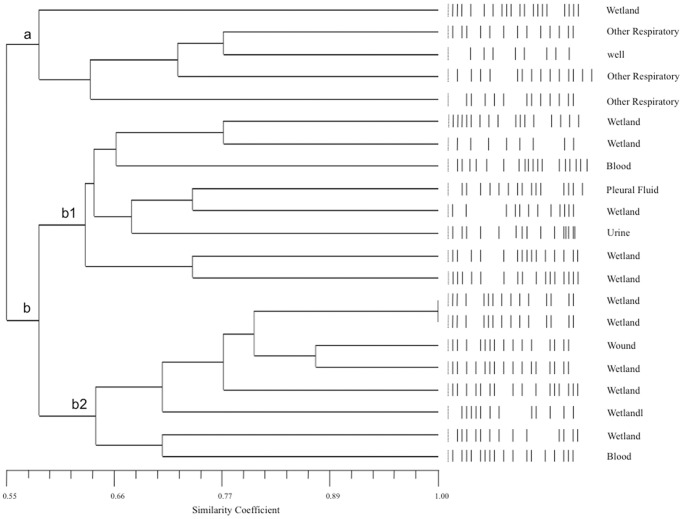
UPGMA dendrogram based on the PFGE patterns of *E. faecalis* isolates from clinical samples (blood, pleural fluid, urine, wounds, and other respiratory sites) and water samples (wells, wetland and water treatment plant). The UPGMA dendrogram was constructed with the PFGE patterns of 21 strains using the NTSYS-pc program and Jacquard’s coefficient. Mantel test r = 0.75389, p = 1.0.

**Figure 4:**
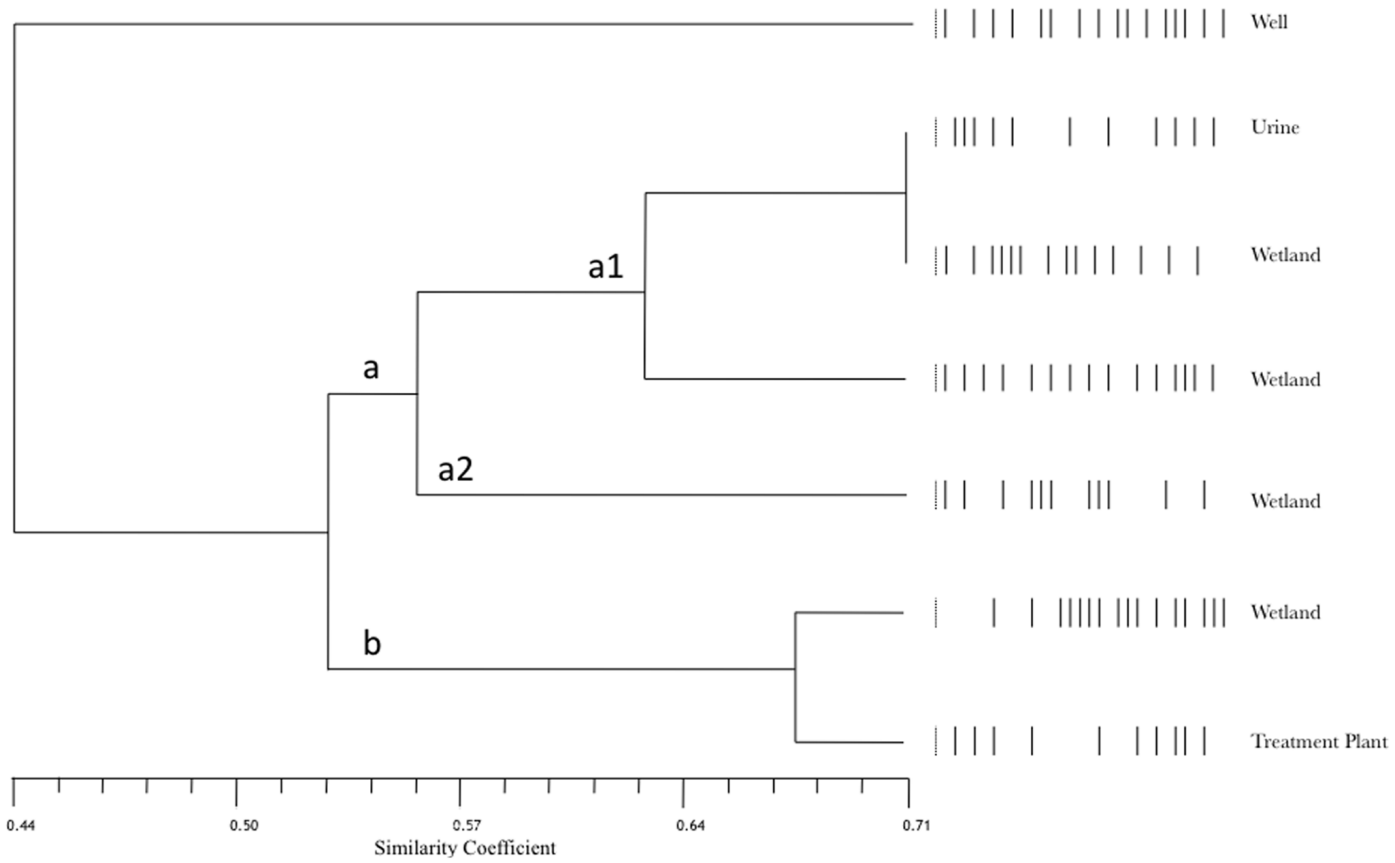
UPGMA dendrogram based on PFGE patterns of *E. faecium* isolates from clinical samples (urine) and water samples (wells, wetland and water treatment plant). The UPGMA dendrogram was constructed with the PFGE patterns of 7 strains using the NTSYS-pc program and Jacquard’s coefficient. Mantel test r = 0.76432, p = 0.999.

From among the 102 strains identified as *E. faecalis* and *E. faecium*, 29 isolates were chosen randomly. Among these, nine (32%) corresponded to clinical strains (eight *E. faecalis* and one *E. faecium*) isolated from blood, pleural fluid, urine, wounds, and respiratory sites. Among the 19 (68%) environmental strains, six were *E. faecium* and 13 were *E. faecalis* isolated from wells, the wetland, and the water treatment plant. The comparison of the PFGE patterns with respect to the average level of diversity for both *E. faecalis* and *E. faecium* (Figures 2, 3 and 4).

Among the *E. faecalis* isolates, the clinical and water samples did not fall into a homogeneous group (Figure 3). Instead, the water isolates had a similarity coefficient greater than 0.50 and fell into two groups (a and b). Clinical isolates from respiratory samples formed the smallest group, which also included a wetland isolate and a water well isolate. The similarity of the clinical respiratory tract isolates (similarity coefficient, 0.77) to the well water isolates (for water for human use and consumption) suggests that there might be a closer association between clinical isolates and well water isolates than between clinical isolates and wetland water isolates.

The water isolate group b formed two distinct subgroups (b1 and b2) related by a similarity coefficient of 0.60. The b1 subgroup also contained clinical isolates from pleural fluid, urinary tract and blood samples, and subgroup b2 included wound and blood isolates. Two strains from the wetland water were shown to have identical PFGE patterns (coefficient of similarity = 1). The two strains with this PFGE pattern were grouped with a wound isolate by a similarity coefficient greater than 0.85, which was the closest association found between a clinical isolate and a water isolate, suggesting that these wetland water strains were related to the *E. faecalis* strain that caused a wound infection. Other isolates in the b2 subgroup were clustered with wetland water isolates with similarity coefficients above 0.70, indicating genetic proximity and suggesting that wetland water isolates could represent a genetic pool of *E. faecalis* strains with the potential to cause infections in hospitalized patients (Figure 3).

Among the *E. faecium* isolates, the isolate with the most distinct PFGE pattern was isolated from a water well and had similarity coefficients under 0.50 with all of the other strains (Figure 4). The rest of the isolates formed a group with PFGE patterns having similarities above 0.50 and consisting of two subgroups a and b. One urine isolated within a1 subgroup was grouped with two strains from the wetland and shared a similarity of 0.71 with one of them, which was the highest similarity coefficient obtained in this analysis (Figure 4). An isolate from the treatment plant water grouped with other isolates from the wetland water but formed a distinct b subgroup. It appears that one isolate among the wetland isolates is closely genetically related to the parental isolate of this treatment plant water isolate. In this study, no association was observed between the *E. faecalis* and *E. faecium* strains with respect to either their PFGE patterns or their antimicrobial susceptibility patterns.

## Discussion

Enterococci identification was performed using the MicroScan gram-positive panel. This system has been previously evaluated using a broad distribution of enterococcal species and showed very good identification of the most common species of enterococci [17], [18], [19]. When d'Azevedo *et al*. evaluated an upgraded version of this system, they found that the system performed well for the identification of *E. faecalis* and typical *E. faecium* [20]. However, sometimes the identification of strains of *E. faecium* was problematic when trying to distinguish them from strains of *E. gallinarum* [21], and similarly, these authors reported errors when identifying strains of *E. durans*, *E. avium, E. raffinosus, E. hirae,* and *E. mundtii* [20]. However, this system has not been tested extensively with environmental isolates.

To overcome these limitations, methods based on the analysis of bacterial DNA have been successfully applied [22], [23], [24]. Identification to the species level using PCR with species-specific primers is a valuable method and can replace complex molecular clustering techniques and conventional microbiological tests that are otherwise necessary to identify species that are difficult to distinguish using phenotypic approaches [25], [26], [27], [28]. Multiplex PCR with specific primers is a simple molecular tool that allows the rapid and accurate identification of enterococci. This technique has been used successfully to identify vancomycin-resistant enterococci [25], [29], but it can also be used to identify all other enterococci [14], [30].

The microbial metabolic characteristics of the *E. faecalis* and *E. faecium* isolates were compared with the taxonomic system described by Teixeira *et al.* [1]. We found that only 41.7% (13/17, Table 2) of the *E. faecalis* strains isolated from the water samples produced acid from sorbitol, in contrast to 76.5% of the isolates from the clinical samples. According to data reported by Teixeira *et al.*, it is expected that 80 to 90% of the isolates would be positive. However, the present findings are not surprising because most metabolic identification schemes are based on the biochemical characteristics of clinical isolates. The biochemical patterns of water isolates have not been studied extensively and may be different [1], [10]. We found sorbitol-positive *E. faecium* strains isolated from clinical and water samples, and it has been suggested that these strains are typically associated with dogs [31]. Our result may therefore indicate that dogs kept as pets play an important role in the transmission of *E. faecium* strains to humans and to the environment.

Regarding the *E. faecium* strains isolated from water samples that were able to utilize raffinose, it has been suggested that *E. faecium* isolates obtained from chicken or poultry can be phenotypically classified according to their raffinose utilization, a unique feature that is not present in this microorganism when isolated from other sources [31]. This observation may indicate that the origin of these environmental isolates may be linked to chicken husbandry in the MCMA.

We found no differences in the frequency of isolation of either *E. faecalis* or *E. faecium* between the clinical and water samples. It is important to note that the water samples exhibited greater diversity in *Enterococcus* species, most likely because the sources of contamination included a wide range of animal as well as human sources.

Enterococci isolated from clinical samples had a higher frequency of antimicrobial resistance than those obtained from water [1]. This result is not surprising because exposure to antibiotics is more common in the hospital setting than in the community (although antibiotics were still available over-the-counter in Mexico during the study period).

Because enterococci have been frequently found in water and because these bacteria are an important cause of infections, it is necessary to elucidate the association between human and water isolates. Therefore, 28 enterococci isolates selected based on their sites of origin, season of isolation (rainy or dry), and antimicrobial susceptibility patterns were genotyped by PFGE to determine their clonal relationships. This analysis demonstrated that the isolates of both clinical and environmental origin showed large genetic variabilities [16], presenting both related and unrelated patterns.

The antimicrobial susceptibility patterns and the PFGE patterns did not exhibit any association, in contrast to a previous report that found a clonal relationship between environmental and clinical isolates [32]. This result suggests that nosocomial infections may be of environmental origin or that these microorganisms may be transferred from hospitals to the environment, allowing their dissemination into the community.

The biochemical and antimicrobial susceptibility patterns of the *Enterococcus* isolates from the clinical and environmental samples showed wide variability, suggesting that the origin of water contamination comes from a variety of sources (including, most likely, animal husbandry). However, water could represent a potential route of transmission of multi-resistant bacteria in the community. Further studies are required to confirm these findings.
